# Embodied perception of alleyways in mountain city historic districts: perspectives of young and older adults in Chongqing Ciqikou

**DOI:** 10.3389/fpsyg.2026.1714333

**Published:** 2026-04-15

**Authors:** Cong Gong, Changjuan Hu, Mingxun Ding, Hao Long, Ying Kang

**Affiliations:** 1 School of Architecture and Urban Planning, Chongqing University, Chongqing, China; 2 Key Laboratory of New Technology for Construction of Cities in Mountain Area, Ministry of Education, Chongqing University, Chongqing, China

**Keywords:** alley spaces, embodied perception, historic district revitalization, mountain city, older adults, young adults

## Abstract

Evaluating embodied perception, the interaction between body movement, sensory input, and the environment, is crucial for addressing diverse needs in mountainous historic districts. Unlike flat districts, where research has explored how spatial elements affect different population groups, perception differences across age groups in mountainous areas remain understudied. This pilot study investigated these differences between younger and older participants in various alley types within Chongqing’s Ciqikou historic district, identifying the spatial factors influencing their perceptions. Data were collected from 46 participants using wearable equipment to monitor real-time psychological and physiological responses. Compound box plots, two-way repeated-measures ANOVA, and Spearman’s correlation were applied to analyze perception differences and the relationships between spatial elements and both physiological and psychological perceptions. Significant physiological differences were found between age groups. The stair alley had a stronger impact on perception than flat alleys. Younger participants exhibited higher stress but more positive emotional feedback compared to older participants. Key factors of mountainous environments, such as slope, spatial openness, and specific sounds, primarily influenced physiological changes. A high degree of coherence between auditory and visual landscape elements was observed across the district, with consistent correlations between spatial characteristics and physiological perception and the correlation between physiological and psychological perceptions. The study recommends improving dynamic and static zoning, lookout spaces, and multi-sensory experiences to enhance spatial quality and preserve the historical identity of mountainous cities.

## 1 Introduction

### 1.1 Theoretical context

As China’s urban development shifts toward regenerating existing resources, the government has increasingly prioritized preserving and enhancing historic districts within urban contexts (Duan et al., 2022). This approach is a response to spatial identity crises triggered by rapid urban expansion (Abdillah et al., 2023; Wu et al., 2022). The International Council on Monuments and Sites (ICOMOS, 2022) emphasizes that historic district preservation should extend beyond merely safeguarding physical structures to prioritizing the perceptual experiences and cultural identities of their users. Mountainous urban areas, with their complex topography, terraced spaces at varying elevations, and three-dimensional ecological patterns, often foster a more intimate human–land relationship than flat regions (Chen et al., 2023; Huang, 2017; Hu and Gong, 2023). In these settings, spatial characteristics and human-environment interactions in historic districts are reflected not only in multi-level accessibility and urban regeneration but also in the interplay between multi-dimensional perception and three-dimensional space (Mandai and Pereira de Souza, 2021; Wan and Shen, 2015). As the number and diversity of visitors to these districts increase, along with the complexity of their needs, traditional approaches focusing solely on physical space while overlooking user experiences are increasingly inadequate. Such approaches fail to meet the varied requirements needed to enhance the quality of mountainous spaces (Jiang and Liu, 2024; Lu et al., 2021), resulting in preservation efforts that do not align with actual usage needs, which undermines the sustainable development and regeneration potential of historic districts (Pendlebury et al., 2004). Consequently, there is an urgent need for research on visitor perception mechanisms and revitalization strategies that can address the diverse needs of users within historic districts in mountainous cities (Lu et al., 2021; Zambon et al., 2019).

### 1.2 Mountainous historic districts and perceptual experience

Recent studies have demonstrated that culturally significant urban areas positively affect mental recovery and psychological wellbeing by providing opportunities for social interaction, physical activity, and a sense of community (Finlay et al., 2015; Yin et al., 2023). Historic districts, which share the origins and history of the city’s development with a city’s development, are vital to both the natural and sociocultural ecology of urban environments. These areas play a key role in supporting the psychological wellbeing of both younger and older populations, promoting social interaction and strengthening cultural spatial identity (Hu et al., 2023). Regenerating historic districts from a perceptual optimization perspective not only enhances the stress recovery potential—the capacity of a space or environment to facilitate the reduction of stress and promote relaxation or recovery from stress—of urban spaces (Almen et al., 2020) but also contributes to fostering a sense of identity and reinforcing sociocultural values within these spaces. Recent studies in Mediterranean and North African contexts have examined how older adults perceive complex urban environments, revealing that spatial and meteorological factors significantly influence their physiological responses and wellbeing (Mahia et al., 2025a; Mahia et al., 2025b). These findings highlight the need for human-centric approaches that integrate physiological monitoring with spatial analysis to address the needs of aging populations across diverse urban settings.

Recent research on spatial perception in urban districts has highlighted the importance of examining the psychological and physiological perceptions of various individuals within different alley spaces. Embodied perception is the dynamic interaction of the brain, body movement, posture, and environment. Subsequently, scholars have investigated the relationships between alley spatial composition and embodied perceptions from multiple perspectives, including spatial form, economic development, social security (Sun et al., 2023), and spatial and visual scale (Li T. et al., 2023). Research on embodied perception in historic districts has focused on physiological and psychological benefits (Li X. et al., 2023) and visual quantification (Zuo et al., 2019) has developed evaluation methods for audio–visual perception (Misthos et al., 2023), as well as physiological and psychological perception (Li J. et al., 2023). Studies have also explored visual (Zheng et al., 2024), auditory (Xie et al., 2014), and cultural gene perceptions (Jiang and Liu, 2024), broadening our understanding of how different population groups interact with alley spaces and contribute to the renewal and development of historic districts. Despite these advances, a gap remains in research on spatial perception across different street types in mountainous cities. Moreover, integrating factors such as spatial congestion (Sever et al., 2018), crowd behavior characteristics (Jo and Jeon, 2020), and multi-modal data (Zhang et al., 2022) to examine emotional interactions between different groups and the built environment could further advance the study of physiological and psychological perceptions in the historic districts of mountainous cities.

### 1.3 Embodied perception and methodological advances

Research on spatially embodied perception has used various methodologies to obtain intuitive feedback on participants’ physiological responses using wearable devices (Schnall, 2017). These studies measure a range of physiological indicators, including heart rate (HR) variability (Liu L. et al., 2022), skin conductance level (SCL) (Sun et al., 2023), mean arterial pressure (Yin et al., 2023), electrodermal activity (Yang et al., 2023), respiratory rate (RESP) (Huang S. et al., 2023; Li and Kang, 2019), skin temperature (Liu B. et al., 2019), blood glucose (Deng et al., 2020), and blood oxygen saturation (Zeng et al., 2023). Additionally, electroencephalography (EEG) indicators such as α-EEG and β-EEG are used to assess responses to changes in diastolic pressure, heightened attention, external stimuli (Cui et al., 2023), and negative emotional experiences (Giannakakis et al., 2019). These indicators are valuable for on-site experiments on visual perception (Chen et al., 2016). Eye-tracking indicators, including average pupil diameter (APD) and saccade frequency, are commonly used in urban environmental evaluations in conjunction with survey scales (Cottet et al., 2018). Some studies have also explored the interactions between environmental elements and eye movement behaviors through acoustic–visual integration experiments, leading to the development of targeted strategies (Liu et al., 2020; Pan et al., 2019). In psychological perception research, indicators such as visual aesthetic quality (Grahn and Stigsdotter, 2010; Xiang et al., 2022), tranquility ratings (Liu Y. et al., 2019), sense of place (Jorgensen and Stedman, 2001), and perceived safety (Foster and Giles-Corti, 2008) have been used to capture subjective perceptions across different populations. When combined with tools like the Place Attachment Scale and the Positive and Negative Affect Scale, these indicators facilitate the analysis of relationships between spatial components and emotional responses (Zhang et al., 2022).

In recent years, experimental research on spatially embodied perception has shifted from laboratory simulations to on-site data collection. Compared to virtual reality (VR) technology, on-site human factors data collection offers a more accurate reflection of mountainous environments’ impact on users, allowing for more precise measurements of perceptual changes. This shift toward on-site multimodal methodologies has been increasingly adopted in studies examining physiological and neurophysiological responses to spatial configurations. For instance, Ma et al. (2023) used EEG to link older adults’ thermal comfort with frontal lobe α waves in outdoor spaces, while Mahia et al. (2025a,b) demonstrated how street geometry, elevation, and multisensory factors influence elderly physiological responses in Mediterranean and Algerian contexts. However, this approach faces challenges, such as the limitations of outdoor data collection equipment and the interference of uncontrolled real-world conditions. Despite these obstacles, research on historic districts using VR technology remains highly active (Zhang et al., 2023), with scholars examining soundscape evaluation and design in historical neighborhoods via actual measurements and questionnaires (Xie et al., 2014). On-site experimental methods combining questionnaires, environmental measurement tools, bodily measurement devices, and real-time physiological data monitoring platforms are progressively advancing.

### 1.4 Age-related perceptual differences

Behavioral observations and demographic research have shown that the primary users of Chongqing’s historic district, a mountainous city, are young (aged 18–42) and older adults (60–75; Jiang and Liu, 2024). Previous studies have demonstrated distinct activity preferences between these groups in historic districts (Huang J. et al., 2023). Young adults tend to favor open, commercial street spaces and exhibit higher usage rates of public areas, while older adults’ use of public spaces is more influenced by objective spatial factors such as street width and road gradients (Li Q. et al., 2023). This suggests that the varied and multi-dimensional spatial environments of mountainous cities may distinctly affect the behavioral and perceptual preferences of each group (Yang et al., 2023).

Both psychological and physiological factors drive differences in spatial perception and activity preferences. Aging fundamentally alters environmental experience through declines in physical health, sensory ability, and climate adaptability, which collectively shape how older adults perceive and respond to complex spatial settings. Young adults, generally in better health and with recreational needs, prefer historical alleys that offer social and cultural experiences (Xie et al., 2024). Conversely, older adults’ spatial perceptions are affected by factors such as climate adaptability, physical ailments, and age-related sensory decline (Chang et al., 2020; Copeland, 2020). As people age, their participation in dynamic outdoor activities typically decreases, leading to reduced spatial perception (Hamm et al., 2021; Landais et al., 2020), although their appreciation of urban aesthetics and spatial satisfaction may increase (Nejati et al., 2016; Ode Sang et al., 2016; Van Dillen et al., 2012). Additionally, the social and wellness needs of older adults influence their spatial perception and inform renewal design strategies (Xu et al., 2022). Therefore, investigating the perceptual preferences of both young and older adults in relation to various alley spaces while considering their distinct physiological and psychological characteristics is essential for designing historic district spaces that accommodate diverse age groups.

### 1.5 Research gap and study contribution

Thus, as research on the interaction between diverse user groups and complex environments advances, multi-dimensional quantitative analysis using multi-modal data is becoming an important method for understanding how people engage with historic districts in mountainous cities. While existing studies have focused on laboratory simulations of spatial perceptions in flat urban areas, there is a growing need for on-site research applying behavioral interventions to investigate perceptual preferences and influencing factors in historic districts within mountainous settings (Czyńska and Rubinowicz, 2019; Hu et al., 2023; Zhang et al., 2022). By controlling environmental variables and conducting multiple on-site experiments, this study offers robust findings based on multi-source data. Further, previous studies on embodied perception in mountainous environments are limited, concentrating on sex differences and cognitive distinctions between residents and tourists (Jiang and Liu, 2024; Xu et al., 2020; Xie et al., 2014). The current study is novel in addressing three research gaps: the lack of systematic comparisons of age-related perceptual differences in mountainous historic districts, where complex topography may amplify such variations, particularly focusing on the physiological–spatial–psychological relationships of these age groups; the scarcity of on-site multimodal investigations integrating EEG, physiological, and perceptual data in authentic mountainous settings; and insufficient attention to alley typology—with its variations in slope, width, and enclosure—as a distinctive spatial configuration influencing age-specific perceptions in mountainous historic contexts. The findings provide a theoretical basis for enhancing human-cent red mountainous environments, the spatial organization of alleyways, and the living experience in historic districts. They can also serve as a foundation for future research on the preservation and revitalization of historic districts in mountainous cities.

## 2 Materials and methods

### 2.1 Research questions

**FIGURE 1:**
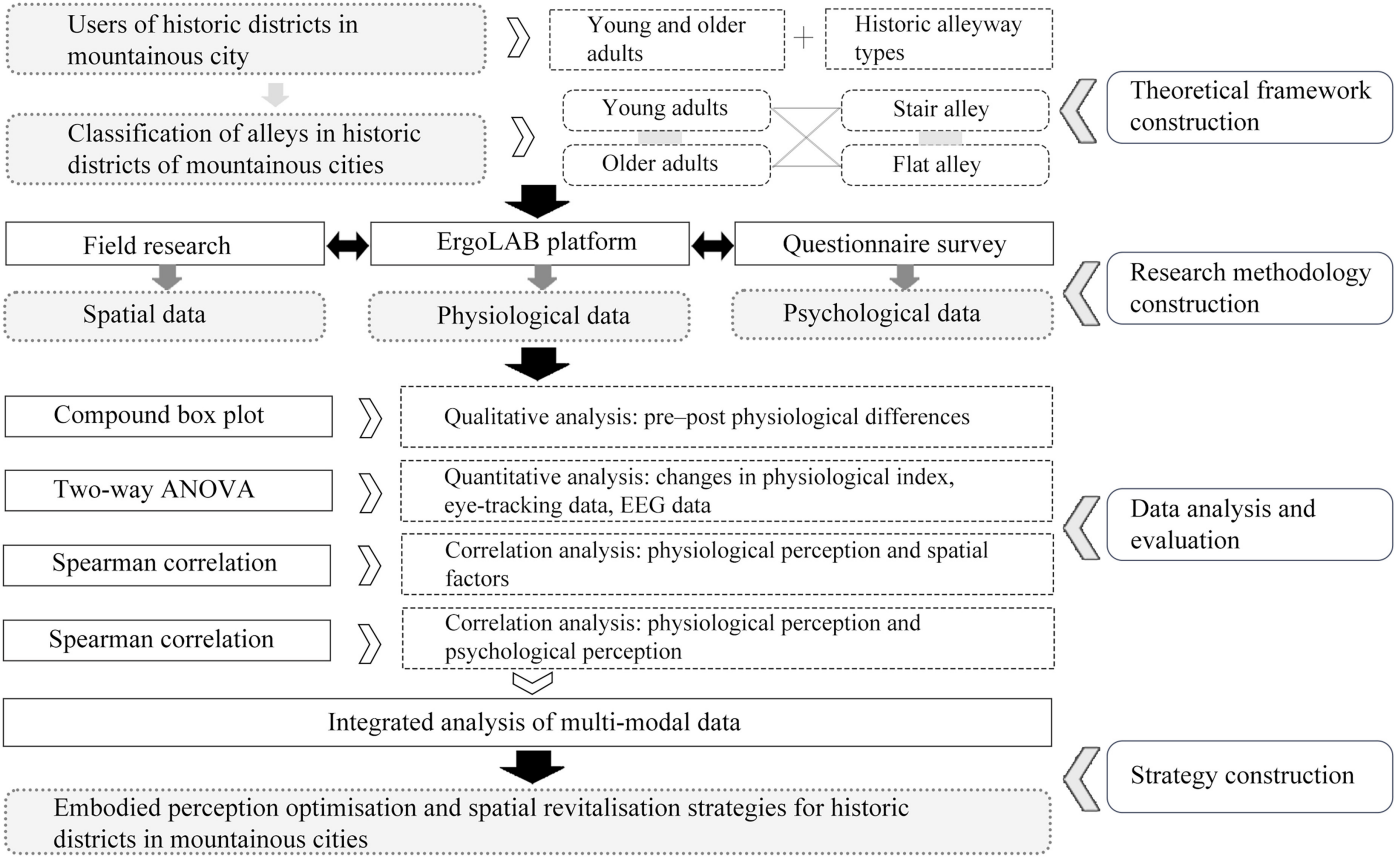
Research framework. ANOVA, analysis of variance; EEG, electroencephalography.

This study employed a within-subject field experimental design to explore causal relationships between spatial factors in historic districts and spatial perceptions across different age groups (see Figure 1). The following research questions were set:

(1) Do the physiological responses of young and older adults differ across various types of historic alleys in mountainous cities? If so, how do these differences manifest?
(2) How do the two independent variables, population group (young vs. older adults) and alley size, as well as their interactions, influence physiological indicators?
(3) What are the impacts of the spatial elements of mountainous historic districts on the physiological indicators of young and older adults?
(4) Is there consistency between the psychological and physiological perceptions of young and older adults across different types of alleys?

### 2.2 Study area

The historic district of Ciqikou, located in Shapingba, Chongqing, China, was selected as the research site. Renowned for its unique geomorphology and distinctive mountainous layout, Ciqikou was designated as one of China’s first historical and cultural districts in 2015. The district reflects the heritage of the Bayu, Shaci, and Hongyan cultures and is a blend of rich historical elements with vibrant recreational and commercial activities.

**FIGURE 2:**
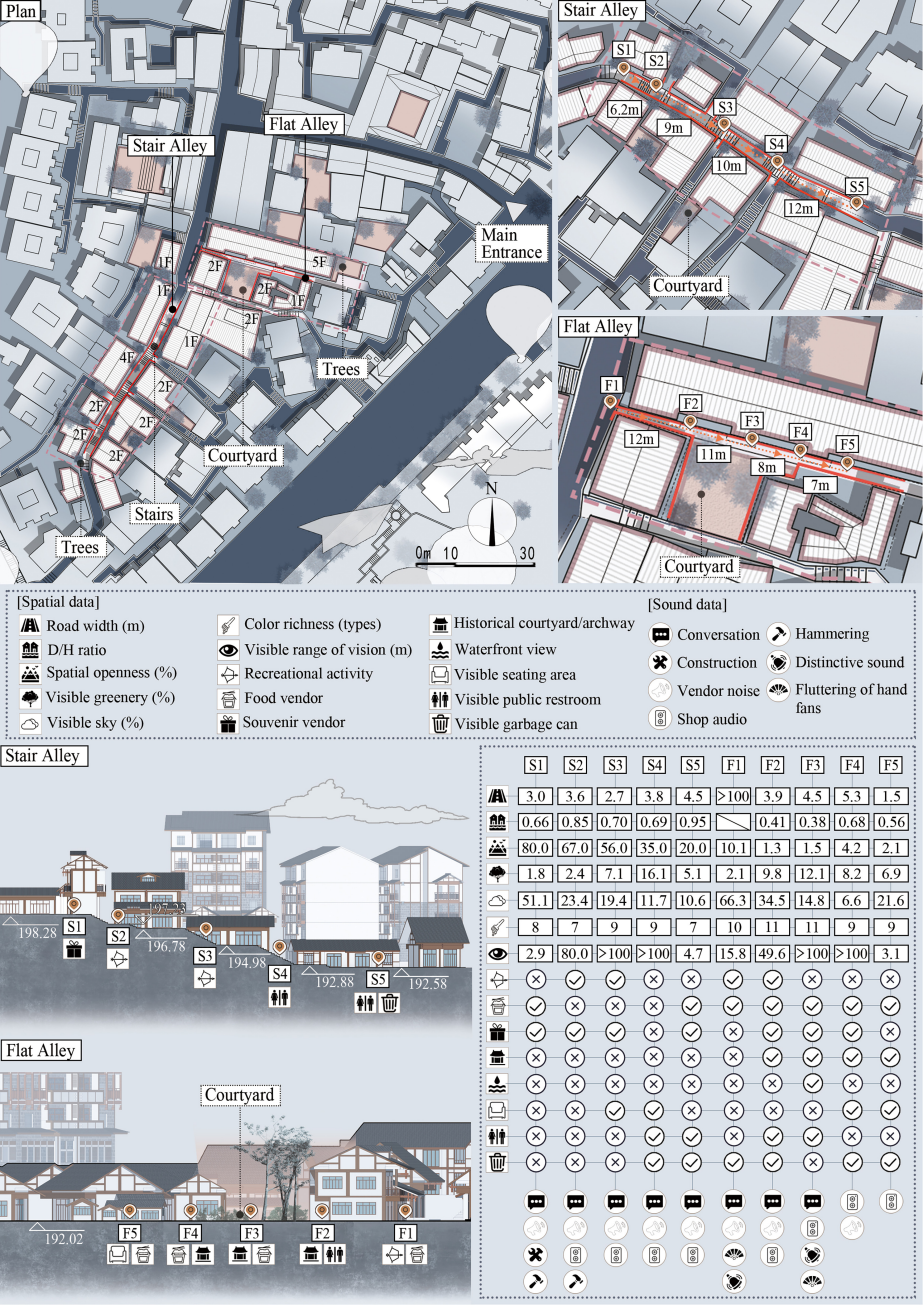
Spatial data of the stair and flat alleys.

Alley spaces in Ciqikou serve as physical manifestations of the complex spatial structures typical of mountainous regions, characterized by multi-level hierarchies, winding pathways, and diverse visual and acoustic elements. Alley typologies were determined through a combined qualitative and quantitative approach. Spatial parameters—including D/H ratio, slope, and openness—were objectively measured using on-site laser distance meters, topographic data, and sky view factor analysis. Commercial intensity was descriptively categorized as moderate, and higher level based on systematic field observations of storefront density along each alley segment. Based on findings from preliminary surveys, semi-structured interviews, and behavioral observations, two main types of alley spaces within the district were selected for the study: stair alley, which highlights the district’s distinct mountainous features, and flat alley, which has historical and commercial significance. For each type of alley, five test nodes were selected, each representing a different set of spatial characteristics (see Figure 2):

- Historic district stair alley (nodes S1–S5): This type is defined by its distinct mountainous terrain, offering unique vantage points and panoramic views. Key spatial characteristics include a staircase pathway, high openness, moderate depth-to-height (D/H) ratio, and a moderate level of commercial types.
- Historic district flat alley (nodes F1–F5): This type represents a historically significant space with a flat pathway, moderate openness, numerous courtyard areas, a lower D/H ratio, and a higher level of commercial types.

Node F1 is a T-shaped intersection where multiple alleyways converge. Unlike linear segments, its width (>100) reflects the open junction space rather than a continuous pathway, which is essential for interpreting its D/H ratio and perception analysis.

Due to the topographical complexity of the mountainous historic district—characterized by steep slopes and extensive stair connections—alley visits followed a fixed spatial sequence based on the actual terrain rather than a randomized order. Consequently, the temporal-spatial sequence within each path was preserved to maintain ecological validity. To minimize carryover effects, 8–10 minute rest intervals were inserted between alley visits, during which participants sat quietly with eyes closed and wore headphones to block visual and auditory stimuli. This duration aligns with evidence that significant physiological changes induced by environmental exposure typically occur within 10 min (Meredith et al., 2020) and is consistent with rest intervals employed in prior on-site studies (e.g., Foellmer and Tyler, 2025).

In this study, embodied perception is conceptualized as a dynamic process involving the real-time interaction between the brain, body, and environment, operationalized through participants’ physiological and psychological feedback during alley exposure. This perceptual process is analytically distinguished from its potential outcomes, which refers to the subsequent changes in psychophysiological states.

### 2.3 Participants and study design

The research team recruited 51 participants on-site who met the following criteria: no history of cardiovascular, psychiatric, or neurological diseases; no use of long-term prescription medications; unaided or corrected visual acuity of 1.0 or better and good sensory abilities (primarily visual and auditory). All participants provided informed consent by signing the “Informed Consent for Perceptual Experiment” in accordance with legal requirements and were informed that their data would be used exclusively for scientific research purposes. The young adult group (18–42 years) was defined following the WHO classification of “youth” (under 44 years). Age was treated as a categorical variable (young vs. older adults) to enable direct comparison between these two primary user groups in mountainous historic districts. To achieve a balanced sex ratio, 27 young adults (18–42; 13 men and 14 women) and 24 older adults (60–75; 12 men and 12 women) were recruited. Participants were instructed to avoid consuming stimulants the day before the experiment and to ensure sufficient rest. After data validation and preliminary analysis, 46 valid samples were obtained: 25 from young adults (13 men and 12 women) and 21 from older adults (10 men and 11 women). The sample size (*N* = 46) was determined a priori using G*Power 3.1, a widely adopted tool in environmental behavior research (e.g., Ruotolo et al., 2024; Perugini et al., 2018), based on an estimated medium effect size (*f* = 0.25), α = 0.05, and power (1–β) = 0.80. A *post-hoc* power analysis conducted on the collected data further confirmed the sample’s adequacy, yielding a power value of 0.9999862, indicating a negligible probability (<0.01%) of Type II error for the significant findings reported.

Alley type and population group were designated as the two independent variables, resulting in four experimental groups: young adults in the stair alley (SY), older adults in the stair alley (SO), young adults in the flat alley (FY), and older adults in the flat alley (FO). A fixed walking route was established for each environment. In the stair alley, the stopping points were designated at the starting and ending points, resting platforms, intersections, and locations with notable changes in the field of view. In the flat alley, stopping points were set at the start and end points, locations with noticeable variations in the D/H ratio, and resting spaces. Each route comprised five key nodes. Participants were instructed to walk at a consistent pace and pause at each stopping point for approximately 15 s while maintaining a view angle between 120° and 180°. Audio data were collected using a PCM-D100 high-fidelity recorder, which was normalized using the Adobe Audition digital audio workstation. To minimize confounding effects from environmental variability across alley spaces, microclimatic conditions were monitored and controlled throughout all on-site experiments. Environmental conditions such as temperature (18–25° C), humidity (60–80%), and light levels (100–1,500 lx) were controlled to ensure accurate perception data.

### 2.4 Measures

A physiological perception indicator system was developed, encompassing three dimensions: electrophysiological, EEG, and eye-tracking (see Table 1). The present study used EEG-α (8–13 Hz), EEG-β (14–30 Hz), and β/α, which are EEG measurements, because they best reflect emotional and stress changes. The corresponding affective indicators assessed included emotion, stress, attention, and cognitive load. Spatial indicators reflecting visitors’ perceptual experiences, such as D/H ratio, slope, commercial types, and openness (Lu et al., 2010), were selected based on the spatial elements of alleys in historic districts of mountainous cities. The unique soundscape features of Ciqikou District include artificial sounds from various businesses, human sounds related to commercial and cultural activities (e.g. yells), and distinctive sounds from intangible cultural heritage (e.g. opera sounds; Xie et al., 2014) were used to create objective soundscape indicators. In addition, spatial congestion characterizes the degree of crowding in a space, which influences behavior (Choi et al., 1976), was considered. Together, these factors constituted eight spatial indicators (see Table 2).

**TABLE 1:** Physiological perception indicators and emotional representations in mountainous historic districts.

Hierarchy	Indicator	Description	Perception
Electro-psychological	Heart rate (HR)	HR indicates that an individual has been stimulated, is under stress, or is in an excited state (Gao et al., 2022).	Emotion
	Heart rate variability (LFa/HFa)	An increased LFa/HFa ratio indicates a state of tension and anxiety, with heightened mental load, often corresponding to negative emotional experiences (Dong et al., 2023).	Stress, emotion
	Respiratory rate (RESP)	An increased RESP indicates a higher degree of emotional engagement; however, it does not allow for the determination of emotional valence (Li and Kang, 2019).	Emotion
	Skin conductance level (SCL)	Elevated SCL can be triggered by states of tension, anxiety, stress, or excitement, indicating higher electrodermal activity (Gao et al., 2022).	Stress,
Electro-encephalography	α-EEG	Increased α-EEG frequency indicates the alleviation of stress and anxiety, while decreased or absent α-EEG frequency suggests enhanced attention or exposure to external stimulus (Cui et al., 2023).	Stress, emotion, attention
	β-EEG	Increased β-EEG frequency is associated with being under stress or in a state of heightened alertness, often corresponding to experiences of negative emotions (Cui et al., 2023). A decrease in β-EEG frequency occurs when attention is dispersed, or emotions improve.	Stress, emotion
	β/α	A higher β/α ratio indicates greater stress, which is often associated with negative emotional experiences (Giannakakis et al., 2019).	Stress
Eye-tracking	Fixation count (FC)	A high FC indicates greater difficulty in element recognition and increased cognitive load, but it does not allow for the degree or valence of emotional association to be determined (Li and Xu, 2009; Zhang et al., 2022).	Cognitive load
	Fixation total duration (FD)	A high FD indicates greater attractiveness and is often associated with positive emotional experiences (Zhang et al., 2022).	Attention, emotion
	Blink count (BC)	High BC indicates increased visual fatigue and relatively dispersed attention (Yan et al., 2024).	Attention
	Average pupil diameter (APD)	Significant changes are associated with emotional dimensions; generally, smaller APD correlates with stronger negative emotions (Zhang et al., 2022).	Emotion

**TABLE 2:** Spatial indicators in mountainous historic districts.

Hierarchy	Indicator	Description
Spatial elements	D/H ratio	D/H ratio refers to the ratio between the distance (D) and height (H) of objects or spatial elements, often applied to assess the human perception of spatial proportion in alleys (Lynch, 1960).
	Slope	Slope refers to the degree of inclination or steepness of a surface. It is a critical factor in mountainous environments and affects accessibility, movement patterns, and overall usability of mountainous areas (Lu et al., 2021; Steiner, 2012).
	Commercial types	Commercial types refer to the various categories or forms of businesses or commercial activities present within the range of the eye-tracker imaging at the node during the stop. It is important to analyze the economic vitality and the social dynamics of a district (Jacobs, 1961).
	Openness	Spatial openness refers to the extent to which a physical space is visually and physically unobstructed, allowing for a clear, expansive view and freedom of movement. It is often used to describe the degree of enclosure or exposure in a built environment (Alexander et al., 1977).
	Spatial congestion	Spatial congestion refers to the level of crowding in mountainous historic districts, typically measured by the density of people within the range of the eye-tracker imaging at the node during the stop (Gehl, 2011).
Soundscape features	Artificial sounds	Artificial Sounds refer to sounds generated by human-made activities or equipment, such as vehicles, machinery, construction, and electronic devices (Kang and Schulte-Fortkamp, 2016).
	Human sounds	Human sounds refer to sounds produced directly by people, such as conversations, footsteps, laughter, coughing, or shouting (Kang and Schulte-Fortkamp, 2016).
	Distinctive sounds	Distinctive sounds refer to unique or characteristic sounds that are specific to a historical environment and distinguish that district from others (Blesser and Salter, 2007).

Based on the perceptual indicators for environmental evaluation proposed by Grahn (1991) and Grahn and Stigsdotter (2010), as well as studies by Xie et al. (2014) and Axelsson (2015), and considering the characteristics of historic districts in mountainous cities, 15 dimensions of visual perception were identified (see Table 3). It should be noted that the “stress recovery potential” aspect is based on stress recovery theory, which posits that pleasurable environments contribute to stress reduction while “serviceability” addresses the varied needs of users over time. For auditory perception, drawing from the International Research Standard on Soundscape (International Organization for Standardization, 2018) and related studies (Axelsson et al., 2010; Axelsson, 2015; Kang et al., 2016), nine dimensions of auditory perception were determined, tailored to the multi-level height differences of mountainous areas (see Table 3). The four overall evaluation indicators incorporate both path node evaluations and the overall environment of mountainous historic districts (see Table 3). Together, these 28 indicators constitute the proposed psychological perception indicator system.

**TABLE 3:** Psychological perception indicators in mountainous historic districts.

Hierarchy	Indicator	Definition
Visual perception indicators	Visual diversity (VD) (Grahn and Stigsdotter, 2010)*	The mountainous visual environment is characterized by rich multi-level layering and diverse land use
	Orderliness (OR) (Grahn and Stigsdotter, 2010)*	The spatial organization of mountainous streets is orderly and of an appropriate scale
	Aesthetics (AE) (Stoltz and Grahn, 2021)	The visual environment possesses certain aesthetic qualities
	Accessibility (AC) (Hu et al., 2024; Yang et al., 2020)*	Pathway connections in mountainous areas are numerous and easily identifiable, with close proximity to entrances and exits
	Comfort (CO) (Ulrich et al., 1991)	The visual environment offers a degree of comfort
	Visual slope (VS) (Yang et al., 2020)*	Elevation differences in mountainous terrain significantly impact visual perception
	Crowding (CR) (Choi et al., 1976)	Crowded pedestrian flow is also a notable aspect of visual perception
	Recognizability (RC) (Hu et al., 2024; Xiao et al., 2023)	The space exhibits a certain level of recognizability and memorable features
	Visual openness (VO) (Kyttä and Kahila, 2005)*	The mountainous visual environment effectively creates open and panoramic spaces
	Publicness (PU) (Gehl, 1987; Xiang et al., 2022)	The environment provides open and accessible space for public activities
	Social (SO) (Grahn and Stigsdotter, 2010; Xiang et al., 2022)	The environment promotes people–environment and people–people socialization
	Refuge (RF) (Grahn and Stigsdotter, 2010; Xiang et al., 2022)*	The mountain environment is safe for walking and personal safety
	Stress relieving (SR) (Li et al., 2021)	The environment is conducive to stress relief
	Serviceability (SE) (Xiao et al., 2023)	The environment provides adequate service facilities
	Cultural (CU) (Li et al., 2021; Xiang et al., 2022)*	The environment contains historical buildings and structures, or historical information and intangible cultural elements
Auditory perception indicators	Pleasant (PL) (International Organization for Standardization, 2018; Kang et al., 2016)	The acoustic environment is pleasant
	Annoying (AN) (International Organization for Standardization, 2018)	The sound environment is irritating
	Calm (CA) (International Organization for Standardization, 2018)	The sound environment is quiet and serene
	Chaotic (CH) (Grahn and Stigsdotter, 2010; International Organization for Standardization, 2018)	The sound environment is chaotic
	Vibrant (VI) (Axelsson et al., 2010)	The sound environment is full of life and vigor
	Monotonous (MO) (International Organization for Standardization, 2018)	The sound environment is monotonous and boring
	Eventful (EV) (Axelsson, 2015; International Organization for Standardization, 2018)*	The acoustic environment is characterized by important events (including historical mountain scenes and industry-specific sounds)
	Uneventful (UEV) (Axelsson, 2015; International Organization for Standardization, 2018)	The sound environment is eventless
	Acoustic diversity (AD) (Grahn and Stigsdotter, 2010; Xie et al., 2014)*	The mountain stereo sound environment is diverse and rich, not monotonous or redundant
Overall perception indicators	Visual environment satisfaction (VES) (Xiang et al., 2022)	Overall perception and evaluation of visual elements
	Acoustic environment satisfaction (AES) (Xiang et al., 2022)	Overall perception and evaluation of auditory elements
	Audio–Visual coherence (AVC) (Hu et al., 2024)	There is acoustic–visual coherence or consistency between auditory and visual environments
	Comprehensive environmental satisfaction (CES) (Hu et al., 2024)	The overall satisfaction of users with historic environments

*Indicators for the most distinctive characteristics of historic districts in mountain cities.

### 2.5 Experimental procedure

The experiment was conducted from 12–25 November 2023. Prior to the experiment, the research team provided participants with a 15-min training video with details on the experimental pathways, equipment usage, precautions, and confidentiality protocols.

Before beginning the experiment, the procedure and precautions were explained to participants. They completed a pre-experiment questionnaire to provide basic information and signed the informed consent form to confirm their understanding of the experimental procedure. After putting on the experimental equipment, they rested for approximately 3 min while the eye-tracking device was calibrated and synchronized with the ErgoLAB platform and eye-tracking video.

**FIGURE 3:**
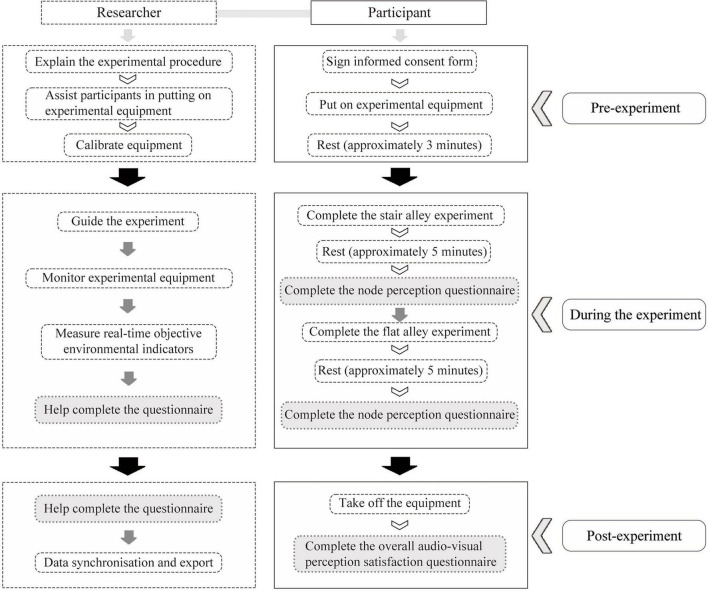
Experimental procedure.

During the experiment, each participant spent approximately 30 min traversing two experimental paths, with each path perception experiment lasting approximately 4–6 min. To minimize errors from testing fatigue, participants rested for 5–8 min between experiments to allow their physiological indicators to return to baseline. They followed a designated route through the historic streets of Ciqikou, stopping at each predefined point for approximately 15 s to survey the surroundings from specified viewpoints. Throughout the experiment, the principal researcher and 2–3 assistants accompanied the participants to ensure that they remained relatively relaxed and minimally disturbed (Figure 3).

**FIGURE 4:**
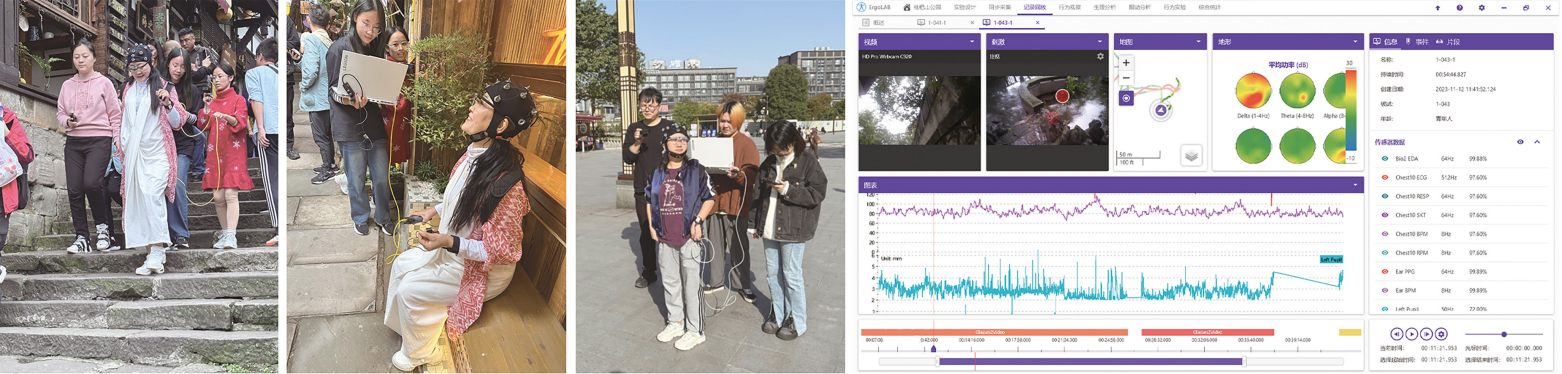
Photos of the experiment.

The principal researcher guided participants down the paths and measured the real-time objective environmental indicators at each stopping point, including the weighted equivalent sound level, temperature, humidity, and light intensity. The researcher also monitored the experimental equipment to ensure accurate and real-time collection of physiological data using devices, including physiological electrical instruments, eye-tracking tools, and an EEG machine. These data were synchronized with the path node information using the ErgoLAB DataLOG APP mobile human factors recording system. After completing each path, the researcher assisted participants in completing the node perception questionnaire. Upon completing the entire walking experiment, participants filled out the overall audio–visual perception satisfaction questionnaire. Subsequently, all physiological data, eye-tracking videos, and behavioral trajectories were integrated and synchronized on the ErgoLAB platform to create a comprehensive dataset (Figure 4).

### 2.6 Statistical analyses

After completing the on-site experiments, the research team assessed the completeness of physiological measurements, eye-tracking recordings, and EEG data. To address motion artifacts during walking-based EEG data collection, multiple filtering strategies were applied. A band-pass filter (0.1–30 Hz) removed low-frequency drift and high-frequency noise, and a 50 Hz notch filter eliminated power line interference. For frequency bands of interest (e.g., alpha: 8–13 Hz), additional band-pass filtering was employed to isolate relevant neural activity. Independent component analysis (ICA) was applied to identify and remove ocular, muscular, and cardiac artifacts, supported by eye-tracking and accelerometer data. Participants with > 40% of data segments rejected due to excessive artifacts were excluded, reducing the sample from 51 to 46. This threshold is consistent with standards in on-site ambulatory EEG research. They calibrated the synchronization between the eye-tracking device and the physiological measurements, organized and entered questionnaire data into the “WJX” online tool for questionnaire data entry, and analyzed the synchronous recordings by segment. A total of 46 valid datasets were analyzed.

Data analysis involved several steps. First, a compound box plot was employed to qualitatively assess differences in physiological perception data between young and older adults across the different alley spaces. Second, a two-way repeated-measures analysis of variance (ANOVA) was used to quantitatively assess differences in physiological indicators across different alley spaces and age groups. Third, Spearman’s correlation analysis was conducted to investigate the relationship between physiological data and various factors associated with historical mountainous alley spaces. Fourth, Spearman’s correlation analysis was performed to examine the correlation between psychological and physiological perceptions to determine the consistency between the psychological and physiological responses of young and older adults.

## 3 Results

### 3.1 Box plot analysis of physiological indicators

**FIGURE 5:**
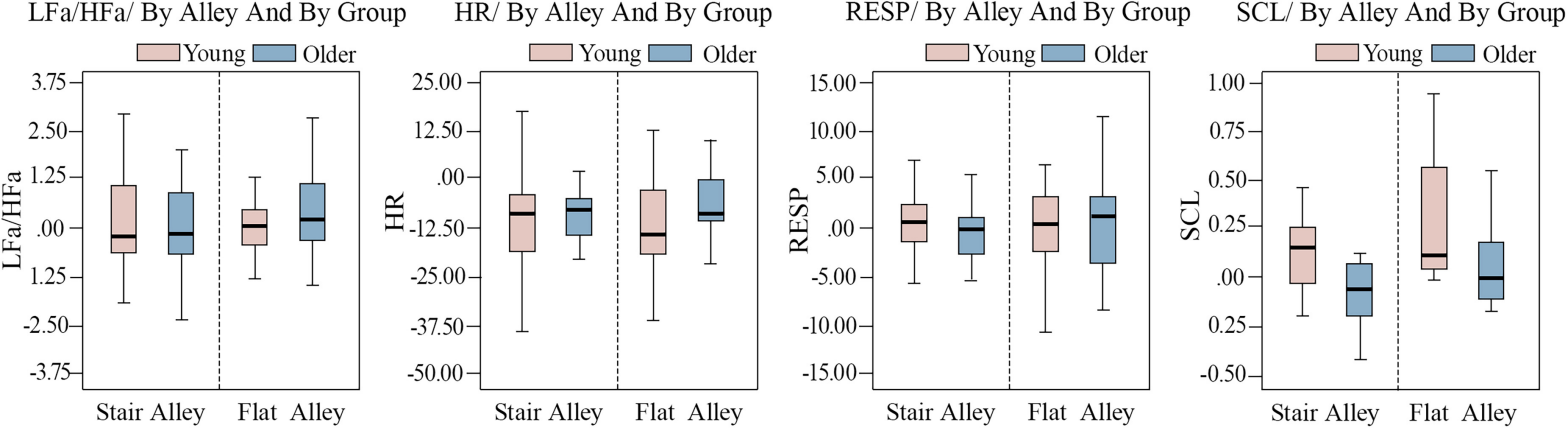
Compound box plots analyzing physiological indicator changes across different alley types and age groups. LFa/HFa, heart rate variability; HR, heart rate; SCL, skin conductance level; RESP, respiratory rate.

The physiological indicators analyzed included HR, HR variability (LFa/HFa), RESP, and SCL. These were statistically analyzed and visualized using compound box plots (see Figure 5). The results revealed that HR changes were lower in the stair alley compared to the flat alley, while changes in LFa/HFa, RESP, and SCL were greater in the stair alley than in the flat alley. In the stair alley, changes in all four physiological indicators were more pronounced in the young group than in the older group, with the SCL indicator showing the most significant difference. In the flat alley, changes in LFa/HFa and RESP were slightly greater in older than in young participants, whereas changes in HR and SCL were significantly greater in younger versus older participants. Based on these results, the following preliminary ranking of physiological perception differences between the two subgroups in different alley types was determined: stair alley > flat alley and young adults > older adults.

### 3.2 T**wo-way** repeated-measures **ANOVA** of physiological indicators

To appropriately address the within-subject design of the alley walks, a two-way repeated-measures analysis of variance (ANOVA) was conducted to examine the main and interaction effects of population group (between-subjects factor: young vs. older adults) and alley type (within-subjects factor: stair vs. flat alley) on physiological indicators. Prior to the analysis, assumption testing was performed; normality was confirmed (*p* > 0.05 for all conditions), and homogeneity of variance was verified (*p* > 0.05), supporting the appropriateness of this statistical approach.

As shown in Table 4, the results indicated that there were no significant interaction effects between the population group and alley type for any of the measured physiological indicators (all p > 0.05). Consequently, the analysis focused on the significant main effects. The between-subjects factor (population group) demonstrated significant main effects on several physiological indicators, including HR (F = 7.153,p = 0.010), SCL (p = 0.028), α-EEG (p = 0.011), β-EEG (p = 0.015), fixation count (FC) p = 0.001, and fixation total duration (FD) p = 0.011. Furthermore, the within-subjects factor (alley type) significantly influenced specific parameters, namely HR F = 4.943, p = 0.031, β-EEG p = 0.040, and average pupil diameter (APD) p = 0.001. The specific variations and descriptive statistics for these main effects are detailed below.

**TABLE 4:** Repeated-measures ANOVA results.

Variable	Effect	*F*	*P*	*p* < 0.05
HR	Group	7.153	0.010	**
HR	Path	4.943	0.031	*
SCL	Group	5.144	0.028	*
α	Group	7.029	0.011	*
β	Group	6.440	0.015	*
β	Path	4.485	0.040	*
FC	Group	13.356	0.001	**
FD	Group	7.123	0.011	*
APD	Path	15.295	0.001	**

Only significant main effects (*p* < 0.05) are displayed. No significant interaction effects (Group × Path) were found for the analyzed indicators. Significance *p < 0.05, **p < 0.01.

Overall, the mean values of the physiological indicators for the young group were higher than those for the older group. Specifically, mean HR, SCL, FC, and fixation total duration (FD) were higher in the younger versus the older group [raw data: HR: 90.04 (Young) (Y) > 83.08 (Older) (O); SCL: 1.76 (Y) > 0.80 (O); FC: 223.81 (Y) > 153.65 (O); FD: 41.49 (Y) > 29.25 (O)]. However, mean α-EEG [raw data: 16.14 (Y) < 20.31 (O)] and β-EEG [raw data: 10.92 (Y) < 14.73 (O)] were lower in the young group than in the older group.

Regarding the differences caused by alley types, both young and older participants exhibited stronger perceptual responses in the stair alley compared to the flat alley. For instance, mean HR was higher in the stair alley [raw data: HR: 89.36 (Stair) (S) > 84.80 (Flat) (F)], whereas mean APD was lower in the stair alley [raw data: 2.90 (S) < 3.29 (F)]. The results also highlighted the impact of different alley types and age groups on physiological responses. Stress-related physiological indicators (i.e., SCL, LFa/HFa, α-EEG, β-EEG, and β/α) were ranked across the four groups (Table 5) as follows: SY > SO > FY > FO. Similarly, when ranking the influence on emotion-related physiological indicators (i.e., HR, LFa/HFa, FC, FD, and APD) across the four groups (Table 5), the ranking of influence was as follows: SY > FY > FO > SO.

**TABLE 5:** Descriptive statistics of physiological indicators (normalized data).

Physiological indicators		Stair alley				Flat alley			
		Young adults		Older adults		Young adults		Older adults	
		Mean	SD	Mean	SD	Mean	SD	Mean	SD
Electro-psychological	LFa/HFa	0.01	0.01	0.07	0.23	0.01	0.01	0.01	0.02
	HR	0.54	0.24	0.42	0.22	0.46	0.23	0.31	0.14
	SCL	0.33	0.34	0.17	0.23	0.32	0.35	0.17	0.23
	RESP	0.73	0.15	0.74	0.15	0.64	0.26	0.71	0.15
Electro-encephalography	α-EEG	0.31	0.16	0.46	0.17	0.37	0.22	0.45	0.17
	β-EEG	0.16	0.14	0.32	0.17	0.29	0.25	0.35	0.20
	β/α	0.20	0.03	0.20	0.02	0.22	0.17	0.20	0.04
Eye-tracking	FC	0.68	0.20	0.43	0.35	0.60	0.16	0.44	0.22
	FD	0.53	0.21	0.35	0.31	0.48	0.17	0.36	0.22
	BC	0.10	0.16	0.20	0.24	0.68	0.58	0.10	0.08
	APD	0.38	0.14	0.36	0.26	0.54	0.17	0.49	0.23

### 3.3 Correlation analysis of physiological and spatial indicators

**FIGURE 6:**
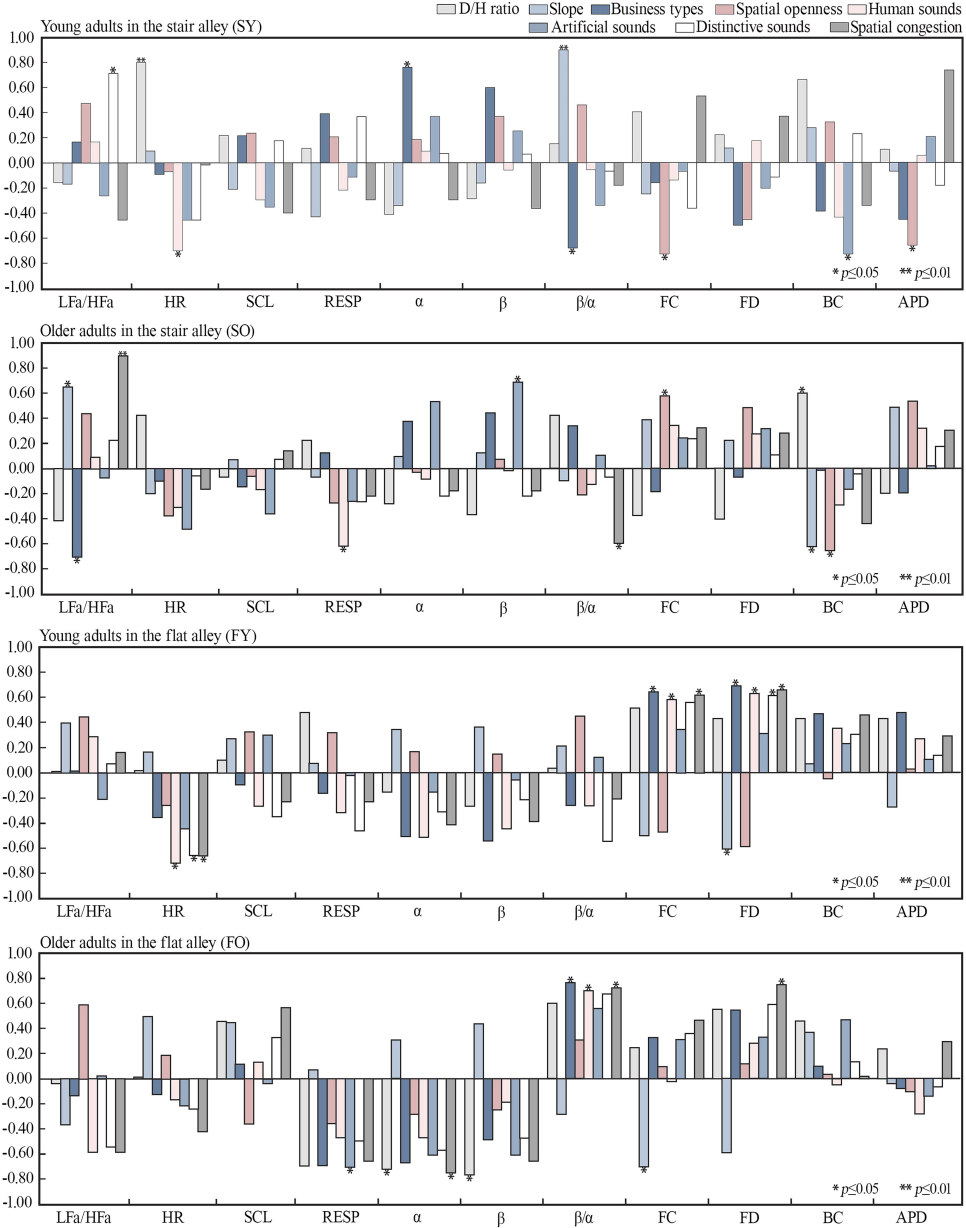
Correlation analysis of psychological and spatial indicators. LFa/HFa, heart rate variability; HR, heart rate; SCL, skin conductance level; RESP, respiratory rate; FC, fixation count; FD, fixed total duration; BC, blink count; APD, average pupil diameter.

To investigate the relationship between physiological responses and spatial elements, and given that these associations were expected to be non-linear, Spearman’s rank correlation analysis was employed to assess the relationships between spatial indicators and psychological/perceptual measures. This analysis was conducted in conjunction with electrophysiological, electroencephalography (EEG), and eye-tracking data, alongside eight spatial indicators (see Figure 6). The results revealed distinct correlations between spatial elements and physiological measures across age groups and alley type. Regarding the stair alley, the β/α ratio for the young group was significantly positively correlated with slope (SY = 0.93, *p* < 0.01), HR was significantly positively correlated with the D/H ratio (SY = 0.80, *p* < 0.01), and FC was negatively correlated with spatial openness (SY = −0.76, *p* < 0.05). For the older group, LFa/HFa was significantly positively correlated with spatial congestion (SO = 0.90, *p* < 0.01) and positively correlated with slope (SO = 0.47, *p* < 0.05). FC was positively correlated with spatial openness (SO = 0.58, *p* < 0.05), and BC was positively correlated with D/H ratio (SO = 0.60, *p* < 0.05) but negatively correlated with slope (SO = −0.61, *p* < 0.05).

Regarding the flat alley, for the young group, HR was negatively correlated with human sounds, special sounds, and spatial congestion (*p* > 0.05). FC was positively correlated with commercial types (FY = 0.70, *p* < 0.05), human sounds (FY = 0.64, *p* < 0.05), specific sounds (FY = 0.62, *p* < 0.05), and spatial congestion (FY = 0.66, *p* < 0.05). For the older group, the β/α ratio was positively correlated with commercial types (FO = 0.78, *p* < 0.05), human sounds (FO = 0.71, *p* < 0.05), and spatial congestion (FO = 0.74, *p* < 0.05). FC was positively correlated with spatial congestion (FO = 0.76, *p* < 0.05), while α-EEG was negatively correlated with the D/H ratio (FO = −0.73, *p* < 0.05). These results suggest that the same spatial elements exert different impacts on the physiological perceptions of young and older populations.

### 3.4 Correlation analysis of physiological and psychological indicators and satisfaction evaluation

**FIGURE 7:**
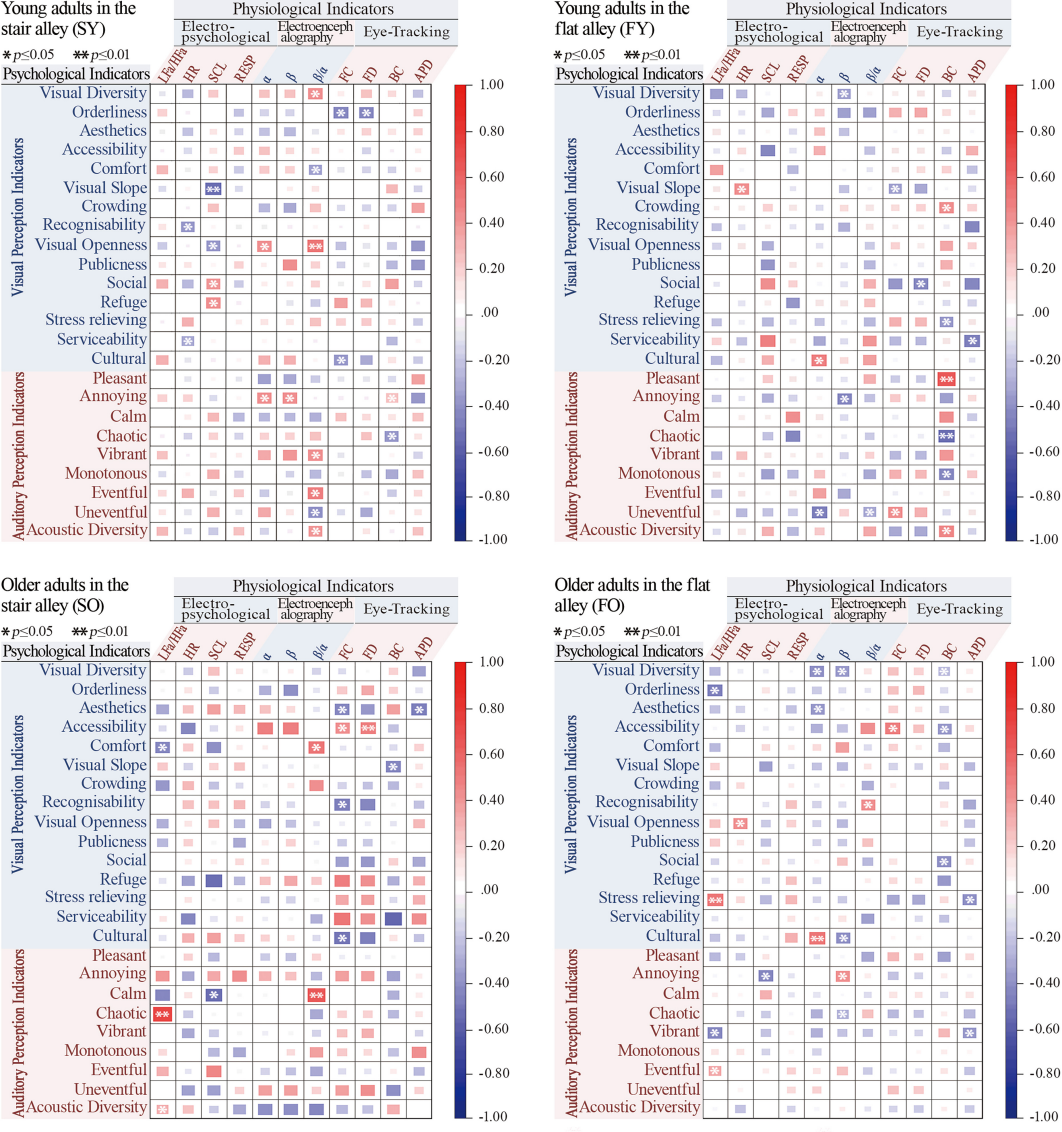
Correlation analysis of physiological and psychological indicators.

**FIGURE 8:**
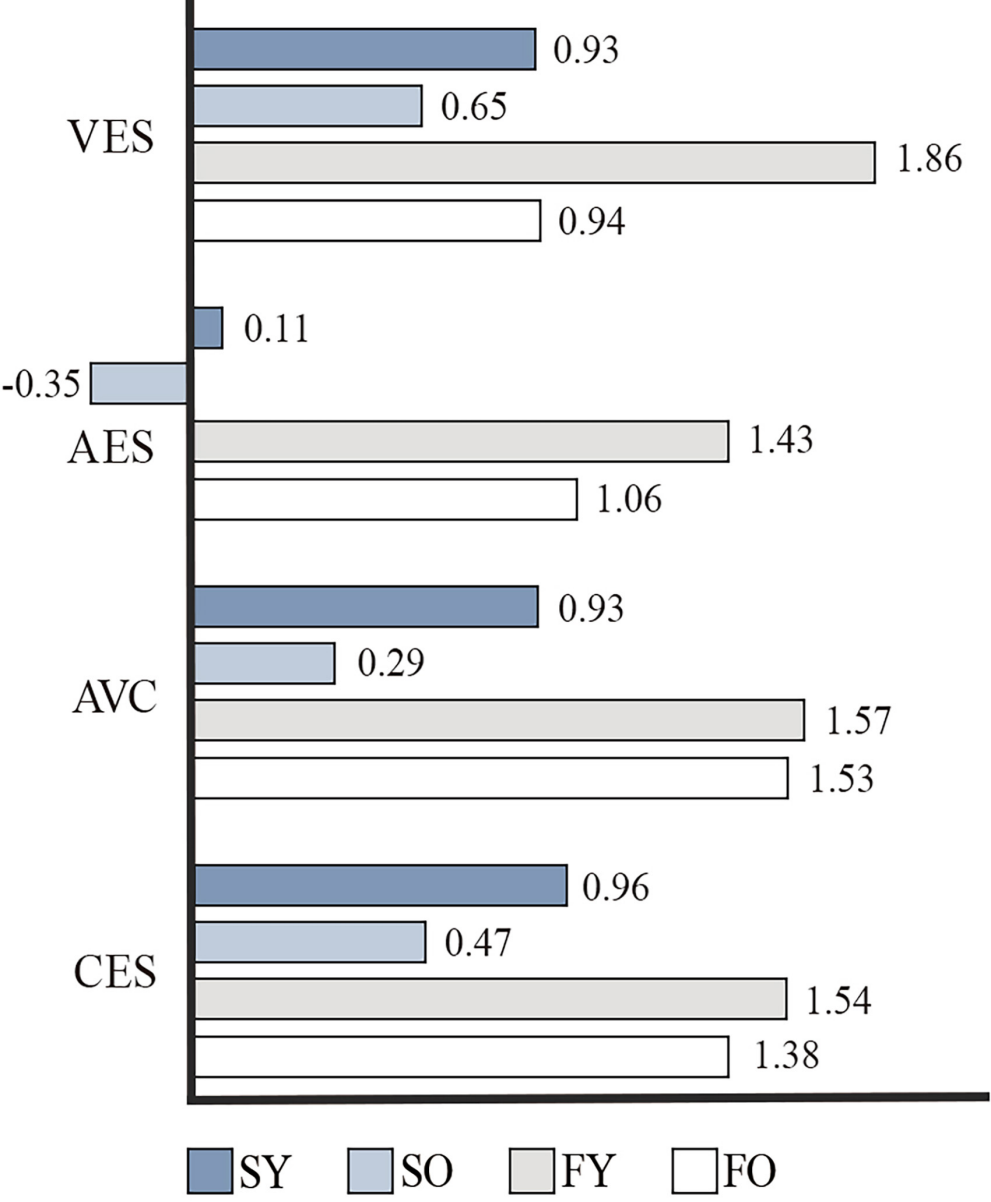
Mean satisfaction analysis results.

**FIGURE 9:**
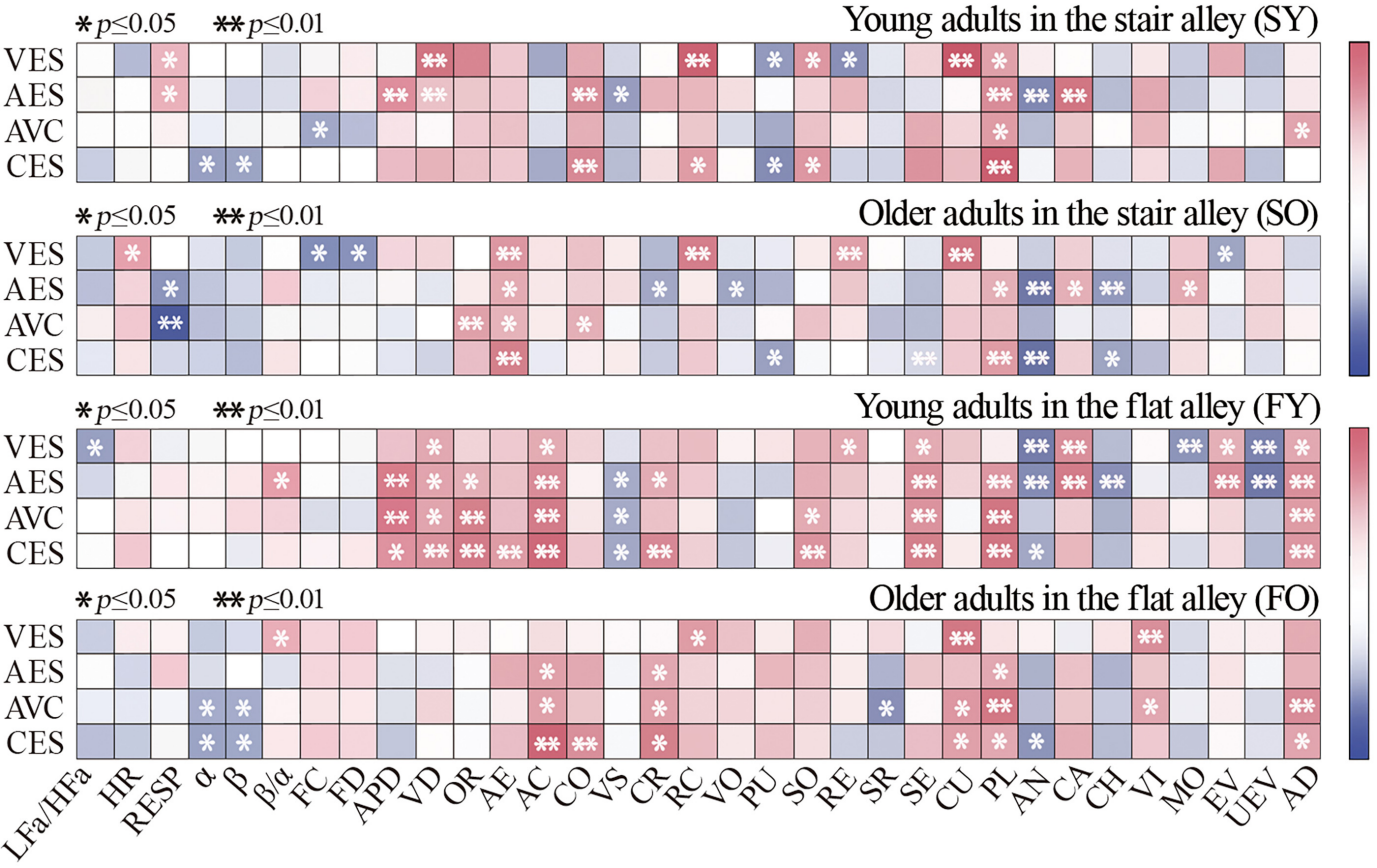
Correlation analysis of satisfaction ratings and psychological and physiological indicators. LFa/HFa, heart rate variability; HR, heart rate; SCL, skin conductance level; RESP, respiratory rate; FC, fixation count; FD, fixed total duration; BC, blink count; APD, average pupil diameter; VES, visual environment satisfaction; AES, acoustic environment satisfaction; AVC, audio–visual coherence; CES, comprehensive environmental satisfaction; FD, fixed total duration; APD, average pupil diameter; VD, visual diversity; OR, orderliness; AE, aesthetics; AC, accessibility; CO, comfort; VS, visual slope; CR, crowding; RC, recognisability; VO, visual openness; PU, publicness; SO, social; RE, refuge; SR, stress relieving; SE, serviceability; CU, cultural; PL, pleasant; AN, annoying; CA, calm; CH, chaotic; VI, vibrant; MO, monotonous; EV, eventful; UEV, uneventful; AD, acoustic diversity.

A total of 46 valid surveys were collected and underwent reliability and validity testing, yielding satisfactory results (KMO = 0.7 [ > 0.6]; Bartlett’s test of sphericity, *p* < 0.001). To explore the correlation between subjective perceptions and objective physical measurements in young and older participants, Spearman correlation analyses were conducted on 11 physiological variables and 24 psychological variables (see Figures 7–9). The analysis showed a high overall audio–visual consistency within the historic district, with visual satisfaction exceeding auditory satisfaction. Younger individuals reported higher overall satisfaction than older adults, and satisfaction ratings for the flat alley were significantly higher than those for the stair alley. The physiological indicators most closely associated with satisfaction were RESP, α-EEG, β-EEG, and BC, while the psychological indicators most closely associated with satisfaction were aesthetics, accessibility, cultural, pleasant, and acoustic diversity.

For the young group in the stair alley, α-EEG was positively correlated with visual openness (SY = 0.42, *p* < 0.05), β-EEG was positively correlated with annoying (SY = 0.43, *p* < 0.05), and the β/α ratio was positively correlated with acoustic diversity (SY = 0.46, *p* < 0.05). For the older group, both FC and FD were positively correlated with accessibility, LFa/HFa was negatively correlated with comfort (SO = −0.58, *p* < 0.05) but positively correlated with chaotic (SO = 0.68, *p* < 0.05), and BC was negatively correlated with visual slope (SO = −0.23, *p* < 0.05).

For the young group in the flat alley, BC was positively correlated with crowding and acoustic diversity (*p* < 0.05), β-EEG was negatively correlated with visual diversity (FY = −0.52, *p* < 0.05), and BC was negatively correlated with monotonous (FY = −0.54, *p* < 0.05). In the older group, FC was positively correlated with visual accessibility (FO = 0.47, *p* < 0.05), α-EEG was positively correlated with cultural (FO = 0.51, *p* < 0.05) but negatively correlated with visual diversity (FO = −0.49, *p* < 0.05), and β-EEG was positively correlated with annoying (FO = 0.44, *p* < 0.05). These results highlight significant differences in both the physiological and psychological perceptions of young and older participants across different alley types.

## 4 Discussion

### 4.1 Physiological variation between young and older adults across different alley types

The compound box plot analysis (see Figure 5) indicated that physiological indicator variations between young and older populations were relatively consistent in the flat alley. However, in the stair alley, the young group exhibited greater variation in physiological responses than the older group. This difference likely stems from age-related disparities in physical capabilities, as the physiological response rate tends to decline with age, resulting in more stable physiological feedback in older adults when navigating the challenging terrain of stair alleys (Copeland, 2020). These findings align with those of Hamm et al. (2021), who observed, through multi-level models, that older adults generally experience higher levels of calmness and lower levels of excitement than young adults. Additionally, the variation in perceptual experiences appeared to be influenced by the distinct environmental characteristics of mountain regions. The results showed that, compared to the flat alley, the stair alley in the historic district had a greater impact on the perceptual experiences of both age groups, with a more pronounced effect on young adults. This is likely because stair alleys offer richer visual stimuli and distinctive spatial qualities, associated with the distinctive mountainous regional characteristics (Liu Z. et al., 2022; Lu et al., 2021). These elements are more likely to capture the interest and attention of young adults, enhancing their overall perceptual experience.

### 4.2 Main effects of age–alley type on physiological indicators

The repeated-measures ANOVA revealed that the overall perceptual experience of young participants was stronger than that of older participants. Specifically, the alleys in the mountainous historic district were more attractive to young adults and elicited higher levels of positive emotions. The young group exhibited a greater visual engagement with the street spaces, including longer gazing times and a higher frequency of fixations, indicating they spent more time perceiving and processing the information of the historic district compared to older adults. This observation aligns with the lower mean values of α-EEG and β-EEG among the young participants, suggesting the young adults were more intensely stimulated by external factors and experienced more positive emotional responses. These findings are consistent with those of Jiang and Liu (2024), who indicated that older adults have significantly lower perceptual engagement than other age groups and tend to perceive historic districts through the lens of cultural belonging and life experiences, whereas young adults are more likely to rely on audio–visual perception.

Regarding the main effect of alley type, both age groups exhibited increased emotional arousal, distraction, and negative emotions in the stair alley versus the flat alley, indicating that both subgroups experienced higher levels of physical load (as opposed to cognitive load) in the stair alley. Long staircases may impose physical strain, particularly on the knees, for both younger and older individuals, which may hinder their ability to fully appreciate the scenery or engage in leisure activities such as shopping (Landais et al., 2020). By contrast, the flat alley, characterized by more historical commercial value, which distinguishes it from other districts, and an easier terrain, tends to enhance visual appeal and reduce stress, thereby mitigating negative emotions among older adults during their walks (Li T. et al., 2023).

### 4.3 Impact of spatial indicators on physiological perception in young and older adults

The correlation analysis between spatial and physiological data within the mountainous historic district revealed that the continuous steps in the stair alley induced stress among young adults. However, as the D/H ratio increased, they displayed a heightened interest in the street environment. This may be attributed to the reduced spatial openness, which increases cognitive engagement by creating more points of interest. These findings are consistent with those of Dai et al. (2022), who noted that young individuals are particularly attracted to environments with pronounced topographical variation and dynamic activities, as these satisfy their inherent desire for challenge and adventure.

By contrast, older adults experienced increased levels of nervousness and anxiety in crowded environments and a higher degree of nervousness and attentiveness in the stair alley versus the flat alley. These results align with those of Landais et al. (2020), who found that older individuals experience greater physiological loads and muscle fatigue on steeper staircases than younger individuals, along with significantly different gait patterns. These findings underscore the stress associated with navigating extended staircases. To mitigate these effects, older individuals require more rest spaces, positive environmental stimuli, and enhanced gait stability, particularly in crowded stair alleys.

In the flat alley, the findings suggest that the abundance of human-related sounds, distinctive sounds, and commercial types attracted young individuals but simultaneously increased their cognitive load. This is consistent with the findings of Khalifa et al. (2022), who observed that monotonous, highly sheltered environments evoke negative emotions in young adults. Introducing interactive spaces in alleyway revitalization could effectively reduce stress in young adults. Conversely, older adults experienced less stress in the flat alley than in the stair alley. However, in the flat alley, increased commercial activity, human sounds, and spatial congestion caused higher stress levels in the older adult group, although a smaller D/H ratio, which offered a sheltered space and a sense of security, helped mitigate these effects. This finding supports the work of Zhang et al. (2022) and Hu et al. (2023), who indicated that older individuals prefer environments that provide both physical shading and a sense of security.

Overall, specific characteristics unique to mountainous environments, such as the D/H ratio, slope, spatial openness, and specific sounds, significantly influenced participants’ physiological responses, primarily through stress, cognitive load, and attention.

### 4.4 Differences in physiological and psychological indicators and satisfaction ratings

Correlation analyses between physiological and psychological data revealed notable differences for the stair alley. For young adults, increased visual openness had a stress-relieving effect, whereas cluttered or layered soundscapes tended to increase their stress levels. By contrast, older adults found visual accessibility and the recognizability of terraced entrances and exits more attractive, which enhanced their spatial recognition. The perceived visual comfort for older adults highlights the importance of lookout spaces created by the mountainous terrain, which help mitigate the mental burden caused by chaotic and irritating soundscapes. These findings align with those of Yao et al. (2022) and Xu et al. (2020), who noted that providing open spaces for older adults in stair alley environments can alleviate the negative emotions associated with navigating staircases.

For the flat alley, the correlation analysis results were broadly consistent with satisfaction evaluations, with satisfaction scores for the flat alley higher than those for the stair alley. For young adults, crowded streetscapes and complex soundscapes were distracting, whereas a diverse array of shops contributed to a sense of comfort. Conversely, older adults considered the historic appearance of streets to be stress relieving, consistent with recent studies suggesting that high-quality historic streetscapes can enhance the sense of security and wellbeing, relieve stress, and promote psychological health in older adults (Nejati et al., 2016; Van Dillen et al., 2012). However, older adults perceived the complexity of shop layouts and the chaotic soundscape as stressful.

Overall, the audio–visual perceptions of both young and older adults were significantly influenced by the distinctive spatial characteristics of the mountainous historic district. There was high audio–visual coordination in this setting, with younger individuals reporting higher overall satisfaction compared to older adults. Although the correlations between mountain spatial features and physiological perception, as well as that between physiological and psychological perception, varied, they led to similar conclusions. For instance, in the stair alley, young adults experienced reduced stress owing to the increased visual openness, whereas greater spatial openness lowered cognitive load and simplified the recognition of elements; both forms of openness made the environment more comfortable and relaxing. Conversely, older adults experienced more stress in the stair alley, as indicated by the positive correlation between LFa/HFa and spatial slope, suggesting the physical strain of climbing a stair alley induces stress. The negative correlation between visual slope and BC, along with the positive correlation between FD and FC, suggests that the stair alley environment heightened concentration and, thus, cognitive load for older adults. However, rich spatial features such as elevated viewpoints and a diversity of shops also made the environment more engaging and comfortable.

### 4.5 Limitations and practical applications

This study has several limitations that could be addressed by future research. First, as the experiment was conducted in a real historic district, external factors that could not be controlled could have affected participants’ physiological and psychological responses. Notably, wireless signal interference severely impacted the accuracy of EEG and physiological measurements, necessitating multiple trials to obtain reliable data. Second, the site’s status as a tourist attraction meant that varying visitor flow at different times of the day may have affected data collection. Although the experiment was mostly conducted between 7:00 and 10:00 am daily to minimize crowd density, this variable should be considered more fully in future studies. Third, although 8–10 min rest intervals were inserted between alley walks to minimize acute carryover effects, participants completed all walks within a single session. This was primarily due to on-site practical constraints, including equipment battery life, borrowing time limits, and potential environmental interference. Fourth, while the older participants were tourists, some younger participants were college students aged 18–22 years, which may not adequately represent the broader spectrum of young adults with diverse social backgrounds, potentially limiting the results’ generalizability. Had the sample included more working professionals or parents—who may experience occupational fatigue, childcare responsibilities, or distinct daily stressors—their physiological and emotional responses to historic alley environments might have differed from those of students, potentially altering the observed patterns of stress recovery and affective feedback. Future research should recruit more diverse young adult subgroups and extend investigations to older adult populations to enable more comprehensive age-comparative analyses. Besides, the broad age range of the young adult group (18–42 years) may mask within-group perceptual variations. Future research could treat age as a continuous variable or use narrower cohorts to examine more fine-grained age-related differences. Lastly, only two alleys typical of mountainous historic districts were investigated, which may not completely capture the full diversity of spatial characteristics in this setting.

Nonetheless, the significance and potential applications of this study are evident. First, the on-site study offers valuable insights by integrating spatial elements with multi-dimensional perceptual data from historic district alleys. It thus provides a theoretical foundation for designing mountain habitats, organizing district spaces, and revitalizing mountainous historic districts. The findings can guide future research on urban development in such cities. Second, by using real-world psychological and physiological data, the study overcomes the limitations of traditional static experiments in controlled environments. It highlights the importance of on-site research in mountainous cities, offering a new preliminary method for studying audio–visual perception in historic districts. Lastly, the findings on embodied perception can inform urban renewal projects. Architects and urban planners can improve the integrated audio–visual environment of historic districts by considering the needs of both younger and older populations, improving spatial quality through evidence-based design.

## 5 Conclusion

This study explored how different alley types in mountainous historic districts impacted the physiological and psychological responses, as well as the overall environmental evaluation of young and older users, and examined the correlations between spatial elements, physiological perception, and psychological factors. Through a comparative analysis of the four experimental groups, the following conclusions can be drawn.

First, significant differences were observed in how young and older adults perceived the two alley types. The stair alley had a greater overall impact on the perceived experience of both age groups than the flat alley, with younger participants showing more variation in physiological indicators, particularly SCL. Second, both stress perception and positive emotional feedback were stronger in young adults than in older adults. While no significant differences were found in stress or positive emotional perception between the young and older populations, the stair alley had a greater impact on stress and positive emotional perception in the young group versus the older group. Third, spatial factors such as the D/H ratio, slope, spatial openness, and specific sound characteristics in the mountainous environment were the primary factors affecting physiological responses in both young and older individuals (Grinfeder et al., 2025). The influence of these spatial indicators varied significantly between the two age groups. Further, the correlation analysis between physiological and psychological perceptions revealed that cultural perception provided stress relief for older adults, while auditory perception had a more significant impact on physiological responses. Fourth, audio–visual consistency in the historic district was high. Young adults reported higher satisfaction levels than older adults, and satisfaction values for the flat alley were higher than those for the stair alley. The integration of mountain-specific spatial data with physiological perception and the correlation between physiological and psychological perceptions varied but ultimately led to consistent conclusions.

Based on the above conclusions, we propose several strategies to optimize the comprehensive perceptual experience of users of historic districts in mountainous cities, fostering more liveable and vibrant alley spaces. First, we recommend optimizing the dynamic and functional zones of alleys. By integrating the unique topography of mountainous terrains with diverse commercial types, alleyways and nodes can be designed to meet young adults’ needs for social interaction and exploration. Combining cascading terraces with commercial spaces can also create resting and viewing areas that meet older adults’ needs for shelter, safety, and tranquility. Second, the functionality of lookout spaces and public infrastructure should be improved. This involves optimizing the connections between stair alley entries and exits, utilizing shop doors that can be completely opened and closed, and linking semi-open spaces like eaves with stair alleys. By coordinating spatial layers, these interventions can improve public facilities, thereby creating more interactive, multi-leveled alley spaces. Third, the revitalization of mountainous historic districts should integrate the cultural diversity of the region with its historical spatial elements, which would boost cultural distinctiveness and prevent the dilution of the area’s unique identity (Acheampong and Asabere, 2021). By considering both visual and acoustic factors in design, multi-sensory environments can be created to improve the overall user experience, reinforcing the historical identity of the space while adapting to contemporary urban life (Duan et al., 2022; Wu et al., 2022).

## Data Availability

The original contributions presented in the study are included in the article/supplementary material, further inquiries can be directed to the corresponding author.

## Glossary

AC (Accessibility): Pathway connections in mountainous areas are numerous and easily identifiable, with close proximity to entrances and exits.
AD (Acoustic diversity): The mountain stereo sound environment is diverse and rich, not monotonous or redundant.
AE (Aesthetics): The visual environment possesses certain aesthetic qualities.
AES (Acoustic environment satisfaction): Overall perception and evaluation of auditory elements.
AN (Annoying): The sound environment is irritating.
APD (Average pupil diameter): Significant changes are associated with emotional dimensions; generally, smaller APD correlates with stronger negative emotions.
Artificial sounds: Artificial sounds refer to sounds generated by human-made activities or equipment, such as vehicles, machinery, construction, and electronic devices.
AVC (Audio–visual coherence): There is acoustic–visual coherence or consistency between auditory and visual environments.
BC (Blink count): High BC indicates increased visual fatigue and relatively dispersed attention.
CA (Calm): The sound environment is quiet and serene.
CES (Comprehensive environmental satisfaction): The overall satisfaction of users with historic environments.
CH (Chaotic): The sound environment is chaotic.
CO (Comfort): The visual environment offers a degree of comfort.
Commercial types: Commercial types refer to the various categories or forms of businesses or commercial activities present within the range of the eye-tracker imaging at the node during the stop. It is important to analyze the economic vitality and the social dynamics of a district.
CR (Crowding): Crowded pedestrian flow is also a notable aspect of visual perception.
CU (Cultural): The environment contains historical buildings and structures, or historical information and intangible cultural elements.
D/H ratio: D/H ratio refers to the ratio between the distance (D) and height (H) of objects or spatial elements, often applied to assess the human perception of spatial proportion in alleys.
Distinctive sounds: Distinctive sounds refer to unique or characteristic sounds that are specific to a historical environment and distinguish that district from others.
Embodied perception: This refers to the capacity of an environment or space to facilitate psychological and physiological recovery from stress.
EV (Eventful): The acoustic environment is characterized by important events (including historical mountain scenes and industry-specific sounds).
FC (Fixation count): A high FC indicates greater difficulty in element recognition and increased cognitive load, but it does not allow for the degree or valence of emotional association to be determined.
FD (Fixation total duration): A high FD indicates greater attractiveness and is often associated with positive emotional experiences.
HR (Heart rate): HR indicates that an individual has been stimulated, is under stress, or is in an excited state.
Human sounds: Human sounds refer to sounds produced directly by people, such as conversations, footsteps, laughter, coughing, or shouting.
LFa/HFa (Heart rate variability): An increased LFa/HFa ratio indicates a state of tension and anxiety, with heightened mental load, often corresponding to negative emotional experiences.
MO (Monotonous): The sound environment is monotonous and boring.
Openness: Spatial openness refers to the extent to which a physical space is visually and physically unobstructed, allowing for a clear, expansive view and freedom of movement. It is often used to describe the degree of enclosure or exposure in a built environment.
OR (Orderliness): The spatial organization of mountainous streets is orderly and on an appropriate scale.
PL (Pleasant): The acoustic environment is pleasant.
PU (Publicness): The environment provides open and accessible space for public activities.
RC (Recognizability): The space exhibits a certain level of recognizability and memorable features.
RESP (Respiratory rate): An increased RESP indicates a higher degree of emotional engagement; however, it does not allow for the determination of emotional valence.
RF (Refuge): The mountain environment is safe for walking and personal safety.
SCL (Skin conductance level): Elevated SCL can be triggered by states of tension, anxiety, stress, or excitement, indicating higher electrodermal activity.
SE (Serviceability): The environment provides adequate service facilities.
Slope: Slope refers to the degree of inclination or steepness of a surface. It is a critical factor in mountainous environments slope affects accessibility, movement patterns, and overall usability of mountainous areas.
SO (Social): The environment promotes people–environment and people–people socialization.
Spatial congestion: Spatial congestion refers to the level of crowding in mountainous historic districts, typically measured by the density of people within the range of the eye-tracker imaging at the node during the stop.
SR (Stress relieving): The environment is conducive to stress relief.
Stress recovery potential: the capacity of a space or environment to facilitate the reduction of stress and promote relaxation or recovery from stress.
UEV (Uneventful): The sound environment is eventless.
VD (Visual diversity): The mountainous visual environment is characterized by rich multi-level layering and diverse land use.
VES (Visual Environment Satisfaction): Overall perception and evaluation of visual elements.
VI (Vibrant): The sound environment is full of life and vigor.
VO(Visual Openness): The mountainous visual environment effectively creates open and panoramic spaces.
VS (Visual Slope): Elevation differences in mountainous terrain significantly impact visual perception.
α-EEG: Increased α-EEG frequency indicates the alleviation of stress and anxiety, while decreased or absent α-EEG frequency suggests enhanced attention or exposure to external stimulus.
β/α: A higher β/α ratio indicates greater stress, which is often associated with negative emotional experiences.
β-EEG: Increased β-EEG frequency is associated with being under stress or in a state of heightened alertness, often corresponding to experiences of negative emotions. A decrease in β-EEG frequency occurs when attention is dispersed, or emotions improve.
